# Cultural adaptation and psychometric evaluation of the Chinese version of the nurse-specific end-of-life professional caregiver survey: a cross-sectional study

**DOI:** 10.1186/s12904-021-00725-2

**Published:** 2021-02-16

**Authors:** Zhijie Zou, Jinbing Bai, Yaohua Gu, Qihua Zou, Canhua Xiao, Jiong Yang, Qing Zhang, Mark Lazenby

**Affiliations:** 1 Department of Respiratory and Critical Care Medicine, Zhongnan Hospital of Wuhan University, Wuhan, Hubei China; 2 Wuhan University School of Health Sciences, Wuhan, Hubei China; 3 Emory University Nell Hodgson Woodruff School of Nursing, Atlanta, GA USA; 4 Yale University School of Nursing, 400 west Campus Drive, Orange, CT USA; 5 University of Connecticut School of Nursing, Storrs, CT USA

**Keywords:** Palliative and hospice care, Chinese version, Nurse, Educational needs, Reliability, Validity

## Abstract

**Background:**

Nurses’ palliative and hospice care-specific education is associated with the quality of palliative and hospice care that influences health outcomes of patients with life-limiting illnesses and their caregivers. However, China lacks measures available to assess nurses’ educational needs in palliative and hospice care. The End-of-Life Professional Caregiver Survey (EPCS) is a psychometrically reliable self-reporting scale to measure multidisciplinary professionals’ palliative and hospice care educational needs. This study was performed to explore the psychometric properties of the Chinese version of the EPCS (EPCS-C) among Chinese nurses.

**Methods:**

We translated and culturally adapted the EPCS into Chinese based on Beaton and colleagues’ instrument adaptation process. A cross-sectional study design was used. We recruited 312 nurses from 1482 nurses in a tertiary hospital in central China using convenience sampling to complete the study. Participants completed the EPCS-C and a demographic questionnaire. Exploratory and confirmatory factor analysis was carried out to test and verify the construct validity of the nurse-specific EPCS-C. Cronbach’s alpha coefficient was used to appraise the reliability of the nurse-specific EPCS-C.

**Results:**

A three-factor structure of EPCS-C was determined, including cultural, ethical, and national values; patient- and family-centered communication; and effective care delivery. The exploratory factor analysis explained 70.82% of the total variances. The 3-factor solution of the nurse-specific EPCS-C had a satisfactory model fit: χ2 = 537.96, χ2/df = 2.96, CFI = 0.94, RMSEA = 0.079, IFI = 0.94, and GFI = 0.86. Cronbach’s alpha coefficient of the overall questionnaire was 0.96.

**Conclusions:**

The nurse-specific EPCS-C showed satisfactory reliability and validity to assess nurses’ palliative and hospice care educational need. Further research is required to verify the reliability and validity of the EPCS-C in a larger sample, especially the criterion-related validity.

**Supplementary Information:**

The online version contains supplementary material available at 10.1186/s12904-021-00725-2.

## Background

Every year, over 56.8 million people in the world are estimated to require palliative care [1]. With a population of more than 1.4 billion [2], China has a huge population in need of palliative care. In 2015, the Quality of Death Index ranked China 71/80 in quality of death and 69/80 in quality of palliative care [3]. Thus, China needs a large workforce with palliative care education and training to provide quality palliative care and improve the quality of death through hospice.

Over the past 25 years, awareness and expansion of palliative and hospice care in China has improved. However, nurses still lack education and training on palliative and hospice care [4–6]. One investigation showed that over 60% of nurses in hospitals of a major Chinese city had never heard of palliative and hospice care [5]. To decrease the gap in this area, the National Health and Family Planning Commission of the People’s Republic of China released detailed guidelines on palliative and hospice care, which include basic standards and management standards for hospice center (trial) [7] and practice guidelines for hospice care (trial) [8]. These guidelines have spawned educational efforts (trainings, workshops, conferences, and courses) for professional palliative and hospice care providers, in particular for nurses, to satisfy their education needs and to enhance their knowledge, attitudes, and self-efficacy of delivering palliative and hospice care. However, there is a paucity of standardized and validated instruments in Chinese to measure nurses’ palliative and hospice care educational needs and self-efficacy.

The End-of-Life Professional Caregiver Survey (EPCS - see Additional file 1) is a self-reporting scale to measure multidisciplinary professionals’ palliative and hospice care educational needs [9–12]. The EPCS was developed in the United States [9] and covers all 8 domains as suggested by American National Clinical Practice Guidelines for Palliative care [13, 14] and the modules of the End-of-Life Nursing Education Consortium (ELNEC) curricula [15, 16]. This EPCS has been widely used in United States [10, 11] and has been tested in other cultural contexts [12, 17, 18]. The EPCS has good psychometric properties, with the Cronbach’s alpha of whole scale and each dimension reported by Lazenby and colleagues as 0.96, 0.95, 0.89, and 0.87 [9]. Until now, China has lacked measures available to assess nurses’ specific educational needs in palliative and hospice care.

## Methods

### Aim

The aim of this study was to translate and culturally adapt the EPCS to access the palliative and hospice care educational needs among Chinese nurses. We reported the translation, cultural adaptation, and psychometric evaluation of the Chinese version of the EPCS (EPCS-C).

### Design and setting

The cross-sectional study was implemented on 57 units of a Level A tertiary hospital in central China. The hospital employs 1542 nurses. The data collection was completed between November 2018 and January 2019.

### Participants

Nurses were eligible for the study if they were currently working as a registered nurse in China with at least one year of experience and the ability to complete questionnaires in Modern Standard Chinese (Mandarin). Nurses who were in training as new nurses were excluded.

Nunnally and Bernstein’s psychometric theory [19] of 5–10 participants per item was adopted to guide the sample size calculation. With 28 items in the EPCS, the estimated sample size would be between 140 and 280. Taking into account a projected 85% completion rate, the total sample size was 329 in this study.

### Developing the Chinese version of nurse-specific EPCS

The EPCS consists of 28 items representing three factors: 12-item Patient- and Family-Centered Communication (PFCC) factor; 8-item Cultural and Ethical Values (CEV) factor; and 8-item Effective Care Delivery (ECD) factor. A 5-point Likert scale ranging from 0 to 4 was used to scale each item. Adding all items’ scores produces the total score, ranging 0–112. A higher total score suggests fewer educational needs.

We obtained the English version of the EPCS from the official website and acquired the permission from the original developer (ML) to translate it into a Chinese version. We translated and culturally adapted the EPCS into a Chinese version based on Beaton and colleagues’ cross-cultural adaptation process [20]. Two bilingual (Chinese and English) nurse scientists (YG & ZZ) independently performed the forward translation. A third nurse scientist (XP) synthesized both forward-translated versions into one Chinese version and arbitrated disagreements until the translators reached consensus that the forward-translated EPCS-C was consistent with the original one from the perspective of language and culture. Using the final forward-translated, another bilingual (native Chinese and near-native English) translator (SC) back-translated the Chinese version into English. The original developer of the questionnaire (ML) and a monolingual English-speaking advanced practice palliative and hospice nurse (CE) compared the back-translated EPCS with the original one and made suggestions. Then, four translators (YG, ZZ, XP, SC) and a monolingual Chinese-speaking palliative and hospice nurse (LF) modified and obtained the pre-final EPCS-C by reaching consensus on semantic, habitual, experiential, and conceptual equivalence of all items.

The pre-final EPCS-C was tested on 24 nurses who came from the Level A tertiary hospital and fit inclusion criteria. Nurses read the pre-final version of the EPCS on paper printout. Then a research assistant interviewed them to ask which items were unclear or not understandable and recorded their answers and suggestions for rewording. From these interviews, misunderstood words and phrases were identified. Items were changed as necessary to form the final Chinese version of the nurse-specific EPCS, which was then used for validation. Three items were reworded after investigators conducted interviews to assess clarity and understandability.

### Data collection

Nurses working in an affiliated hospital of Wuhan University were recruited by electronic advertisements which briefly stated the study’s aim and procedure. The electronic advertisements together with an online questionnaire link made by Wenjuanxing [21], a professional online questionnaire survey, evaluation, and voting platform, were sent to unit managers. This link allowed nurses to view the informed consent form and choose whether or not to agree to participate in anonymous surveys. Only after the nurse clicked the option of being aware of the informed consent and agreeing to take part in in the survey would the page jump to the questionnaire page. The questionnaire page included a general information questionnaire developed by researchers and the EPCS-C. The survey would end if the agreeing to participate option was not clicked. A total of 329 questionnaires were collected, of which 15 were removed because the participants had not been worked in nursing for more than one year and 2 were removed because of too much missing data. Thus, the final number of samples to be analyzed was 312.

### Data analysis

All the data were analyzed using SPSS 22.0 and Amos 17.0. Two-tailed significance tests were performed with a *p* value of 0.05 as the significance level. Participant demographic characteristics were described using mean (standard deviation), frequencies (percentage), and measures of central tendency and dispersion.

We used a 3-step process to validate the nurse-specific EPCS-C.

**Step 1: Content Validity.** Content validity was assessed in the translation and adaptation of the EPCS-C and was established with 100% agreement among investigators.

**Step 2: Internal Consistency**. Cronbach’s α coefficient was calculated to appraise the internal consistency of total scale and each subscale. We considered a value of 0.6 or higher for each subscale and a value of 0.7 or higher for the whole scale as adequate internal consistency [22, 23].

**Step 3: Construct Validity.** Exploratory factor analysis (EFA) and confirmatory factor analysis (CFA) were used. Prior to performing EFA, we conducted Kaiser-Myer-Olkin (KMO) test and Bartlett’s test to ascertain the factorability [24]. The EFA was used to seek the factor structure of nurse-specific EPCS-C and provide information for generating a modified factor solution. The number of factors reserved to rotation was determined through an Eigenvalue ≥1, the scree plot, and the explainability of the different factor solutions generated. The item loading of factors were extracted by the principal component analysis with varimax rotation. Then, the CFA was carried out to certify the construct validity of the proposed modified factor solution through computing standardized factor loadings and model fit indices. A loading value of 0.4 or more for a factor means that the item can be attributed to the underlying factor [25]. Model fit indices, involving model χ2, χ2/df, comparative fit index (CFI), incremental fit index (IFI), root-mean-square error of approximation (RMSEA), and goodness-of-fit index (GFI), were applied to evaluate the goodness of fit of the model. If χ2/df ≤ 3, CFI > 0.90, RMSEA< 0.06, IFI > 0.90, and GFI > 0.90, the model fitting was admissible [25]. Hu and Bentler suggested that the model had a fair fit if RMSEA was between 0.05 and 0.08 [26].

We analyzed the influence of age, length of work in year, school education on palliative and hospice care, and the related training at work on participants’ scores on the nurse-specific EPCS-C using Spearman Correlation and liner regression analyses.

## Results

### Participants

The participants’ general information are shown in Table 1. The majority of participants were female (92.9%) and had a bachelor’s degree in nursing (89.7%). The mean age of participants was 28.96 ± 5.78 years. On average, they worked as a nurse for 7.2 ± 6.4 years. Most participants (82.4%) had experience of caring for a dying patient, while fewer (22.1%) had cared for a dying patient within the last month.

**Table 1:** Characteristics of participants (*N* = 312)

	Mean (SD)	Range	n (%)
Age, year	28.96 (5.78)	21–50	
Sex			
female			290 (92.9)
male			22 (7.1)
Marital status			
married			174 (55.8)
divorced			1 (0.3)
single			137 (43.9)
Highest academic degree			
technical secondary school			1 (0.3)
junior college/Diploma			24 (7.7)
Bachelor of Science in Nursing			280 (89.7)
Master of Science in Nursing			7 (2.2)
Religious belief			
no			295 (94.6)
buddhism			14 (4.5)
christianity			1 (0.3)
others			2 (0.6)
Working as a nurse, year	7.25 (6.42)	1–31.42	
Title			
nurse			83 (26.6)
senior nurse			178 (57.1)
supervisor nurse			50 (16)
associate professor of nursing			1 (0.3)
Position			
staff nurse			304 (97.4)
head nurse			7 (2.2)
nursing division manager			1 (0.3)
School education on palliative care	0.95 (0.85)	0 (none) - 3 (sufficient)	
Training at work on palliative care	1.16 (0.90)	0 (none) – 3 (sufficient)	
Cared for a dying patient			
yes			257 (82.4)
no			55 (17.6)
The latest time of caring a dying patient			
within 1 week			33 (10.6)
within 1 month			66 (21.2)
within 3 months			59 (18.9)
within 6 months			28 (9.0)
within 12 months			71 (22.8)

### Construct validity

The KMO value of the nurse-specific EPCS-C was 0.964 and Bartlett’s test was highly significant (*p* < 0.001). Thus, EFA was conducted. After conducting the principal component analysis with Varimax rotation [25], three factors emerged with a cumulative variance of 70.71%, including 14-item Factor 1 (eigenvalue 16.6; variance 59.1%); 6-item Factor 2 (eigenvalue 2.1; variance 7.4%); and 8-item Factor 3 (eigenvalue 1.2; variance 4.1%). All item loadings were > 0.50. Four items (6, 7, 8, 14) were removed due to high cross-loadings in two factors (both > 0.50).

The KMO value of the 24 items was 0.96 and Bartlett’s test was highly significant (*p* < 0.001). The new three-factor model emerged with a cumulative variance of 71.60%, including 14-item Factor 1 (eigenvalue 14.41; variance 60.06%); 5-item Factor 2 (eigenvalue 1.72; variance 7.18%); and 5-item Factor 3 (eigenvalue 1.05; variance 4.36%). We removed another three original items (19, 23, 24) according to modification indices produced by the AMOS program and the researchers’ judgement.

The final EFA was conducted for the 21-item EPCS-C. The KMO value was 0.96 and the Bartlett’s test was highly significant (*p* < 0.001). The final three factors emerged (Table 2) with a cumulative variance of 70.82%: 11-item Factor 1 (eigenvalue 12.37; variance 58.90%); 5-item Factor 2 (eigenvalue 1.46; variance 6.95%); and 5-item Factor 3 (eigenvalue 1.04; variance 4.97%). According to the item content, we named Factor 1 the Cultural, Ethical, and National Values (CENV), Factor 2 the PFCC, Factor 3 the ECD subscales.

**Table 2:** Loading value from the last EFA for EPCS-C and mean of each item

Factor	Original EPCS Item Number	Factor Loadings			Mean (SD)
		Factor 1	Factor 2	Factor 3	
Cultural, Ethical, and National Values	22	**.844**	.215	.149	1.70 (1.079)
	21	**.800**	.220	.322	1.91 (1.047)
	25	**.798**	.254	.157	1.92 (1.076)
	27	**.790**	.383	.140	1.79 (1.080)
	26	**.761**	.345	.167	1.85 (1.059)
	20	**.754**	.219	.387	1.99 (1.016)
	18	**.738**	.353	.343	2.04 (0.989)
	16	**.728**	.349	.314	1.98 (1.035)
	13	**.636**	.388	.397	2.09 (0.956)
	28	**.600**	.333	.368	2.20 (1.073)
	17	**.577**	.237	.467	2.16 (1.048)
Patient- and Family-Centered Communication	4	.196	**.756**	.166	2.19 (1.149)
	2	.272	**.735**	.366	2.35 (1.015)
	3	.346	**.731**	.325	2.15 (1.052)
	1	.318	**.711**	.220	2.19 (1.005)
	5	.439	**.671**	.206	2.14 (1.062)
Effective Care Delivery	9	.052	.206	**.818**	2.94 (0.903)
	11	.354	.276	**.697**	2.31 (0.976)
	10	.420	.215	**.682**	2.29 (0.943)
	12	.390	.472	**.582**	2.34 (0.985)
	15	.392	.353	**.510**	2.35 (1.026)

Note: Loading values for the same factor are bold

**Fig. 1:**
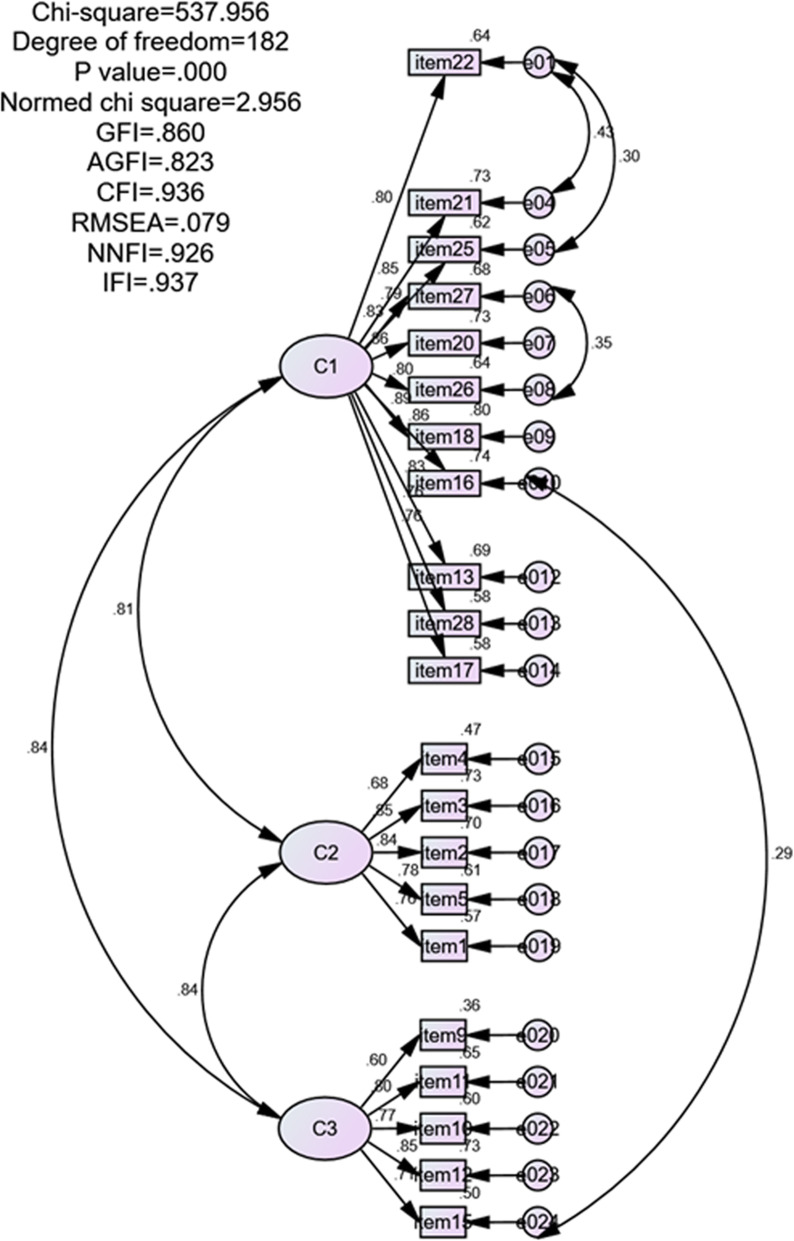
Confirmatory factor analysis of the final proposed model of EPCS-C

The CFA was performed for the final proposed factor solution. According to modification indices, correlations between error variances were allowed to advance model fit [27]. The model fit indices were: χ2 = 537.96, χ2/df = 2.96, CFI = 0.94, RMSEA = 0.079, IFI = 0.94, and GFI = 0.86. The standardized factor loadings for the final model ranged from 0.60 to 0.89 and were all statistically significant (Fig. 1). Thus, a final new 3-factor model was validated, namely the nurse-specific EPCS-C (see Additional file 2) model. Table 3 compares the factor structures between the original model [9] and the nurse-specific EPCS-C model.

**Table 3:** Comparison of the factor structures between the original model and the proposed model

Proposed model (21 items)		Original model (28 items)	
Subscale	items	Subscale	items
PFCC	1,2,3,4,5	PFCC	1,2,3,4,5,6,7,8,9,10,11,12
CENV	13,16,17,18,20,21,22,25,26,27,28	CEV	13,14,15,16,17,18,19,20
ECD	9,10,11,12,15	ECD	21,22,23,24,25,26,27,28

*Note*: *PFCC* patient- and family-centered communication, *CENV* cultural, ethical, and national values, *CEV* Cultural and Ethical Values, *ECD* effective care delivery

### Internal consistency

Cronbach’s alpha coefficients of the 21-item nurse specific EPCS-C and its PFCC, CENV and ECD subscales were 0.96, 0.89, 0.96 and 0.86, respectively (Table 4).

**Table 4:** The Cronbach’s alpha coefficients and score of the EPCS-C

Factor	Cronbach’s alpha coefficient	Mean score Mean (SD)	Mean score per item Mean (SD)
PFCC	0.887	11.02 (4.39)	2.20 (0.88)
CENV	0.956	21.63 (9.66)	1.97 (0.88)
ECD	0.864	12.23 (3.90)	2.45 (0.78)
Total	0.964	44.88 (16.49)	2.14 (0.79)

*Note*: *PFCC* patient- and family-centered communication, *CENV* cultural, ethical, and national values, *ECD* effective care delivery

### Nurses-specific EPCS-C score

The mean total score of the nurse-specific EPCS-C was 44.88 ± 16.49. Nurses scored higher score on the Effective Care Delivery subscale (mean per item = 2.45 ± 0.78) but lower on the Cultural, Ethical, and National Values subscale (mean per item = 1.97 ± 0.88). For individual items, the lowest scoring item was “I am familiar with palliative care principles and national guidelines” (1.70 ± 1.08). (Details are presented in Tables 2 and 4).

School education on palliative and hospice care and the related training at work were associated with the nurse-specific EPCS-C score (Table 5). Marital status, clinical title, whether cared for a dying patient, and the latest time of caring a dying patient were not associated with the EPCS-C score. The regression results showed that training on palliative and hospice care at work was the only one factor that predicted EPCS mean scores (Table 6).

**Table 5:** Spearman correlation coefficients between EPCS-c and Characteristics of participants

	EPCS-C score	
	r	p
Age	−0.050	0.381
Working year	−0.055	0.329
School education on palliative care	0.164	**0.004**
Training on palliative care at work	0.199	**0.000**

**Table 6:** Liner regression on EPCS-C

	B	β	t	p	95.0% Confidence Interval for B
(Constant)	39.640		25.244	**.000**	36.550 ~ 42.730
School education on palliative care	1.255	.065	1.003	.317	−1.207 ~ 3.718
Training at work on palliative care	3.480	.190	2.940	**.004**	1.151 ~ 5.810

## Discussion

The purpose of this study was to translate and culturally adapt the EPCS for use among Chinese nurses. The EPCS is widely used to measure multidisciplinary professionals’ palliative and hospice care educational needs. We followed Beaton et al.’s cross-cultural adaptation process to translate the EPCS into Chinese and tested its psychometric properties among Chinese nurses [20]. Data from a total of 312 nurses were analyzed and results showed that the 21-item nurse-specific EPCS-C exhibits strong internal reliability and construct validity.

Both EFA and CFA were carried out to test and verify the construct validity of the nurse-specific EPCS-C. All the analyses suggested three factors –CENV, PFCC, and ECD, which was consistent with the original EPCS. Based on the CFA, the final nurse-specific EPCS-C showed a satisfactory model fit. Furthermore, the high standardized factor loadings of each subscale verified the construct validity of the nurse-specific EPCS-C. The nurse-specific EPCS-C had high Cronbach’s alpha coefficients on both the whole scale and the three subscales, ranging from 0.86–0.96. Therefore, the EPCS-C is a reliable instrument for assessing palliative and hospice care education needs among nurses [28].

Until now, only the scales testing people’s knowledge of and attitudes toward palliative and hospice care had been translated into Chinese, such as the Palliative Care Quiz for Nursing [6, 29] and Frommelt Attitudes Toward Care Of the Dying Scale [30, 31]. Besides knowing nurses’ exact knowledge of and their attitudes toward palliative and hospice care, the increasing national emphasis on palliative and hospice care in China makes it imperative that we know nurses’ educational needs, to provide tailored training. The nurse-specific EPCS-C is a valid instrument for assessing nurses’ education needs.

However, we did note differences between the EPCS-C and the EPCS, which are common when the factor structure of a scale is tested under different cultural contexts [20, 32]. First, compared to the original 28-item EPCS, the EPCS-C includes 21 items. Some original items were deleted because they were almost equally loaded on 2 factors, making them indistinguishable. Some items were removed according to modification indices achieving by the AMOS program and the researchers’ judgement. Moreover, some items were moved to another factor instead of the original one. In fact, there are not only differences but also connections among the three factors. For example, original item 11 (“I know how to use non-drug therapies in management of patients’ symptoms”) (see Additional file 1) was moved from the PFCC subscale to the ECD subscale. Non-drug therapies include different therapies besides communication, such as musical therapy, mindfulness-based cognitive therapy, meditation and so forth, and providing them is considered effective care delivery. But, the final nurse-specific EPCS-C like the original EPCS covers all domains of the American National Clinical Practice Guidelines for Palliative care [14] and the modules of ELNEC curricula [15].

The study showed that training on palliative care at work was an influential factor in EPCS-C score, which was similar with the literature [10]. As we know, the low quality of palliative and hospice care in China [3], specific palliative and hospice care education should be advocated for the professional caregivers.

The convenience sampling limited the study. Further study is required to verify the psychometric properties of the EPCS-C in a larger sample, especially the criterion-related validity. Other scales testing people’s knowledge of and attitudes toward palliative and hospice care should be used to test their relevance to the EPCS-C. In addition, some misunderstood words and phrases for nurses were found during pilot test, such as advanced care planning, hospice, palliative care, and spiritual issues. In order to collect data more truthfully, we briefly introduced the above words in the questionnaire of this study and suggested that they should be explained in future studies. Furthermore, we only tested the EPCS-C among nurses. Future work needs to be done on other professionals, especially physicians, who are also important team members of palliative and hospice care. Given the scale of end-of-life care needs in Chinese-speaking populations and the gap in trained nurses to meet those needs, that we now have a validated survey to test Chinese-speaking nurses’ educational requirements in palliative and hospice care is a strength of this study.

## Conclusions

The EPCS-C demonstrates sufficient reliability and validity for assessing palliative and hospice care-specific education need among nurses. This scale provides nurse leaders and managers and nursing faculty with an easily administered tool for Chinese-speaking nurses to assess palliative and hospice care-specific educational needs. It also provides researchers with an instrument to explore the effect of education on nurses’ provision of palliative and hospice care.

## Supplementary Information

**Additional file 1.** End-of-Life Professional Caregiver Survey.

**Additional file 2.** Chinese version of End-of-Life Professional Caregiver Survey.

## Data Availability

The data used during this study are available from the corresponding authors on reasonable request.

## Abbreviations

CENV: cultural, ethical, and national values
CEV: Cultural and Ethical Values
CFA: confirmatory factor analysis
CFI: comparative fit index
ECD: effective care delivery
EFA: exploratory factor analysis
ELNEC: End-of-Life Nursing Education Consortium
EPCS: End-of-Life Professional Caregiver Survey
EPCS-C: Chinese version of the EPCS
GFI: goodness-of-fit index
IFI: incremental fit index
KMO: Kaiser-Myer-Olkin
PFCC: patient- and family-centered communication
RMSEA: root-mean-square error of approximation
