# Supplementary material for: Cultural adaptation and psychometric evaluation of the Chinese version of the nurse-specific end-of-life professional caregiver survey: a cross-sectional study
Source: BMC Palliat Care. 2021 Feb 16;20:32. doi: 10.1186/s12904-021-00725-2 (PMC7885229; doi:10.1186/s12904-021-00725-2)
Supplement: Supplementary file 1 — Additional file 1. End-of-Life Professional Caregiver Survey. [file 12904_2021_725_MOESM1_ESM.docx]

End-of-Life Professional Caregiver Survey

Below is a list of statements that other end-of-life professional caregivers have said are important.

**Please circle one number per line to indicate your response as it applies to you today.**

|  |  | Not at all | A little bit | Some- what | Quite a bit | Very much |
| --- | --- | --- | --- | --- | --- | --- |
| P1 | I am comfortable helping families to accept a poor prognosis | 0 | 1 | 2 | 3 | 4 |
| P2 | I am able to set goals for care with patients and families. | 0 | 1 | 2 | 3 | 4 |
| P3 | I am comfortable talking to patients and families about personal choice and self-determination | 0 | 1 | 2 | 3 | 4 |
| P4 | I am comfortable starting and participating in discussions about code status | 0 | 1 | 2 | 3 | 4 |
| P5 | I can assist family members and others through the grieving process | 0 | 1 | 2 | 3 | 4 |
| P6 | I am able to document the needs and interventions of my patients | 0 | 1 | 2 | 3 | 4 |
| P7 | I am comfortable talking with other health care professionals about the care of dying patients | 0 | 1 | 2 | 3 | 4 |
| P8 | I am comfortable helping to resolve difficult family conflicts about end-of-life care | 0 | 1 | 2 | 3 | 4 |
| P9 | I can recognize impending death (physiologic changes) | 0 | 1 | 2 | 3 | 4 |
| P10 | I know how to use non-drug therapies in management of patients’ symptoms | 0 | 1 | 2 | 3 | 4 |
| P11 | I am able to address patients’ and family members’ fears of getting addicted to pain medications | 0 | 1 | 2 | 3 | 4 |
| P12 | I encourage patients and families to complete advanced care planning | 0 | 1 | 2 | 3 | 4 |
| C1 | I am comfortable dealing with ethical issues related to end-of-life/hospice/palliative care | 0 | 1 | 2 | 3 | 4 |
| C2 | I am able to deal with my feelings related to working with dying patients | 0 | 1 | 2 | 3 | 4 |
| C3 | I am able to be present with dying patients | 0 | 1 | 2 | 3 | 4 |
| C4 | I can address spiritual issues with patients and their families | 0 | 1 | 2 | 3 | 4 |
| C5 | I am comfortable dealing with patients’ and families’ religious and cultural perspectives | 0 | 1 | 2 | 3 | 4 |
| C6 | I am comfortable providing grief counseling for families | 0 | 1 | 2 | 3 | 4 |
| C7 | I am comfortable providing grief counseling for staff | 0 | 1 | 2 | 3 | 4 |
| C8 | I am knowledgeable about cultural factors influencing end-of-life care | 0 | 1 | 2 | 3 | 4 |
| E1 | I can recognize when patients are appropriate for referral to hospice | 0 | 1 | 2 | 3 | 4 |
| E2 | I am familiar with palliative care principles and national guidelines | 0 | 1 | 2 | 3 | 4 |
| E3 | I am effective at helping patients and families navigate the health care system | 0 | 1 | 2 | 3 | 4 |
| E4 | I am familiar with the services hospice provides | 0 | 1 | 2 | 3 | 4 |
| E5 | I am effective at helping to maintain continuity across care settings | 0 | 1 | 2 | 3 | 4 |
| E6 | I feel confident addressing requests for assisted suicide | 0 | 1 | 2 | 3 | 4 |
| E7 | I have personal resources to help meet my needs when working with dying patients and families | 0 | 1 | 2 | 3 | 4 |
| E8 | I feel that my workplace provides resources to support staff who care for dying patients | 0 | 1 | 2 | 3 | 4 |
