# Supplementary material for: Cultural adaptation and psychometric evaluation of the Chinese version of the nurse-specific end-of-life professional caregiver survey: a cross-sectional study
Source: BMC Palliat Care. 2021 Feb 16;20:32. doi: 10.1186/s12904-021-00725-2 (PMC7885229; doi:10.1186/s12904-021-00725-2)
Supplement: Supplementary file 2 — Additional file 2. Chinese version of End-of-Life Professional Caregiver Survey. [file 12904_2021_725_MOESM2_ESM.docx]

生命末期疗护医务人员调查

以下为其他生命末期疗护医务人员认为重要的内容。请您仔细阅读每个条目，圈出符合您目前情况的数字。

|  |  | 完全不同意 | 有一点同意 | 有一些  同意 | 比较多  同意 | 非常  同意 |
| --- | --- | --- | --- | --- | --- | --- |
| P1 | 我能自在地帮助患者家属接受患者的不良预后。 | 0 | 1 | 2 | 3 | 4 |
| P2 | 我能同患者和家属一起制定照护目标。 | 0 | 1 | 2 | 3 | 4 |
| P3 | 我能自在地与患者和家属讨论其个人选择和自我决策。 | 0 | 1 | 2 | 3 | 4 |
| P4 | 我能自在地组织和参与是否做心肺复苏术的讨论。 | 0 | 1 | 2 | 3 | 4 |
| P5 | 我能协助患者的家属和其他亲朋好友渡过患者去世后的哀伤期。 | 0 | 1 | 2 | 3 | 4 |
| C1 | 我能自在地处理与生命末期疗护/安宁疗护/缓和疗护有关的伦理问题。 | 0 | 1 | 2 | 3 | 4 |
| C2 | 我可以和患者及其家属一起处理灵性问题。 | 0 | 1 | 2 | 3 | 4 |
| C3 | 我能自在地应对患者和家属的宗教信仰和文化诉求。 | 0 | 1 | 2 | 3 | 4 |
| C4 | 我能自在地为患者家属提供哀伤辅导。 | 0 | 1 | 2 | 3 | 4 |
| C5 | 我熟知影响生命末期疗护的文化因素。 | 0 | 1 | 2 | 3 | 4 |
| C6 | 我能判断患者何时适合转入安宁疗护机构。 | 0 | 1 | 2 | 3 | 4 |
| C7 | 我熟悉缓和疗护的原则和国家指南。 | 0 | 1 | 2 | 3 | 4 |
| C8 | 患者变更疗护地点时，我能有效地帮助其维持疗护的连续性。 | 0 | 1 | 2 | 3 | 4 |
| C9 | 对于患者要求协助其自杀的请求，我有信心处理好。 | 0 | 1 | 2 | 3 | 4 |
| C10 | 疗护临终患者及家属时，我有个人资源帮助我满足自我需求。 | 0 | 1 | 2 | 3 | 4 |
| C11 | 我觉得我的工作单位为疗护临终患者的员工提供了支持资源。 | 0 | 1 | 2 | 3 | 4 |
| E1 | 我能识别濒死（症状和体征）。 | 0 | 1 | 2 | 3 | 4 |
| E2 | 我知道如何使用非药物疗法来管理患者的症状。 | 0 | 1 | 2 | 3 | 4 |
| E3 | 我能消除患者和家庭成员对使用止痛药成瘾的担心。 | 0 | 1 | 2 | 3 | 4 |
| E4 | 我鼓励患者和家属制定预立医疗照护计划。 | 0 | 1 | 2 | 3 | 4 |
| E5 | 我能陪伴在临终患者身旁。 | 0 | 1 | 2 | 3 | 4 |
